# Sensitive timing of undifferentiation in oligodendrocyte progenitor cells and their enhanced maturation in primary visual cortex of binocularly enucleated mice

**DOI:** 10.1371/journal.pone.0257395

**Published:** 2021-09-17

**Authors:** Hyeryun Shin, Hideki Derek Kawai

**Affiliations:** 1 Department of Bioinformatics, Graduate School of Engineering, Soka University, Hachioji, Tokyo, Japan; 2 Department of Biosciences, Graduate School of Science and Engineering, Soka University, Hachioji, Tokyo, Japan; University Hospital Wurzburg, GERMANY

## Abstract

Sensory experience modulates proliferation, differentiation, and migration of oligodendrocyte progenitor cells (OPCs). In the mouse primary visual cortex (V1), visual deprivation-dependent modulation of OPCs has not been demonstrated. Here, we demonstrate that undifferentiated OPCs developmentally peaked around postnatal day (P) 25, and binocular enucleation (BE) from the time of eye opening (P14-15) elevated symmetrically-divided undifferentiated OPCs in a reversible G0/G1 state even more at the bottom lamina of the cortex by reducing maturing oligodendrocyte (OL) lineage cells. Experiments using the sonic hedgehog (Shh) signaling inhibitor cyclopamine in vivo suggested that Shh signaling pathway was involved in the BE-induced undifferentiation process. The undifferentiated OPCs then differentiated within 5 days, independent of the experience, becoming mostly quiescent cells in control mice, while altering the mode of sister cell symmetry and forming quiescent as well as maturing cells in the enucleated mice. At P50, BE increased mature OLs via symmetric and asymmetric modes of cell segregation, resulting in more populated mature OLs at the bottom layer of the cortex. These data suggest that fourth postnatal week, corresponding to the early critical period of ocular dominance plasticity, is a developmentally sensitive period for OPC state changes. Overall, the visual loss promoted undifferentiation at the early period, but later increased the formation of mature OLs via a change in the mode of cell type symmetry at the bottom layer of mouse V1.

## Introduction

Oligodendrocyte progenitor cells (OPCs), also known as NG2-expressing neural progenitor cells (NPCs) or NG2 glia, are the principal proliferative cell type in the postnatal brain and continue to produce newly myelinating oligodendrocytes throughout a lifetime [1–6]. The proliferation and differentiation of OPCs are under the control of neural activity to maintain their optimal cell density and distribution [7]. Stimulation of neuronal circuits up-regulated proliferation and differentiation [8, 9] and leads to thickening of myelin sheath [10] by reactivating quiescent OPCs [11]. Physical exercise reduced proliferation and enhanced differentiation [12], while sensory deprivation by whisker-clipping increased proliferation and lowered differentiation of OPCs in the barrel field of the somatosensory cortex (S1BF) [13, 14].

Evidence for experience-dependent effects on OPC proliferation and differentiation in the primary visual cortex (V1) is lacking. Dark rearing (DR) for 4 weeks did not affect the cell density and distribution of OPCs [13]. However, OPC proliferation and differentiation during the critical period of ocular dominance plasticity (ODP; i.e., P19 to P32 in mice, [15, 16]) may be important to investigate since some myelination associated genes are upregulated at the peak of the critical period [17], particularly in the lower layers [18], and several oligodendrocyte-expressing genes respond to light stimulation in V1 [19, 20]. Although it is unclear if and how myelination-related gene expression is associated with myelin formation, these studies suggest that visual experience during the critical period of ODP could play a role in the oligodendrocyte development, possibly contributing to myelination dependent control of ODP [20].

Here we investigated, in the binocular field of the mouse visual cortex, how oligodendrocyte lineage cells develop, and if and how binocular enucleation alters their proliferation and differentiation. Developmental changes in the cell density of proliferative cells were examined around the onset of the critical period, where we detected not only a developmentally sensitive age of undifferentiation but also elevated undifferentiation of OPCs at the bottom of V1 due to the visual loss. Focusing on the OPCs proliferated for 3 days up to the age, we assessed a visual experience-dependent change in OPC states as well as the symmetric mode of cell division. The proliferated cells were left to develop for 5 and 25 days and examined their states of differentiation. Overall, we conclude that binocular enucleation at the onset of eye-opening induced undifferentiation during the important period of cortical development and later formed more oligodendrocytes via an altered mode of sister cell symmetry.

## Materials and methods

### Animals

Animals were housed in a vivarium with 12hr light/12hr dark cycle. All animal procedures were conducted according to the Guide for the Care and Use of Laboratory Animals [21], and were approved by the Institutional Animal Care and Use Committee of Soka University. All experiments used C57BL/J mice (postnatal days (P) 15–50). All enucleation procedures were based on previous description [22–24] and have been approved by the IACUC (Approval No. 170015, 18013, 190009, 20008, 21006). Individual mice at P15 (the first day of eye-opening) were anesthetized with 2% isoflurane (Cat# 008313, Intervet) with a gas mixture of 1.0 L/min O_2_ and 1.5 L/min N_2_O via a mask over the nose while maintaining the body temperature at 37°C using a heat-pad, and all efforts were made to minimize suffering. After a surgical level of anesthesia to keep them immobilized, we applied 2% lidocaine (Cat# 125–05681, Wako) on and around the eyelid to anesthetize peripheral tissues, the eyelid was opened, the eyeball was lifted away with forceps from the orbit, and optic nerves were occluded along with connected blood vessel for a while then dissected using a surgical scissor. After the enucleation, a piece of sterile Gelfoam (Pfizer) was inserted inside the orbit, the eyelid was closed, and 2% lidocaine or 0.25% bupivacaine (LKT laboratories) was applied to prevent pain locally. An opposite eye was subsequently removed as above. The binocular enucleation procedure typically took under 5 min. For sham control, mice were subjected to the same surgical procedure except the eye removal. Following the surgery, sham control and the enucleated mice were intraperitoneally injected with an analgesia meloxicam (5 mg/kg, Boehringer Ingelheim) daily once for 3 days. We continuously monitored their body temperature, heart rate, and respiration throughout the surgery. The enucleated and sham control mice were returned to their mother and siblings in a cage with nesting materials after surgery. Animals were housed in a vivarium with 12hr light/12hr dark cycle and monitored daily to check the health of the animals. After weaning, mice were hand-reared (typically for 3–5 days) until they could feed off the feeder to ensure survival and minimize the impact of visual compromise. All the animals that undergone surgery survived without significant loss of body weight.

### BrdU and drug administration

For birth timing of cells, each animal was injected intraperitoneally (i.p.) with BrdU (5’-bromo-2-deoxyuridine; Cat# B5002, Sigma) once a day at the dose of 100 mg/kg for 3 days and at 2 hours before sacrifice (~74 hours total) for three different developmental periods: P19-22, P22-25, and P25-28. Mice were perfused with 4% (w/v) paraformaldehyde (Cat# 168–23255, Wako) in 0.1 M phosphate buffer (4% PFA) at the end of each period. Brains were sectioned for immunohistochemistry analysis to determine the cell lineage association with birth time. Cyclopamine (Cat# C-8700, LC Laboratories) was used at 1 mg/ml mixed with HBC (2-hydropropyl-β-cyclodextrin; Cat# H-107, Sigma) prepared as a 45% solution in PBS [25]. Mice were injected daily with HBC alone (vehicle control) or cyclopamine at 25 mg/kg once daily for 4 days from P22-25 or 8 days from P22-29 and sacrificed at P25 or P30, respectively. BrdU was injected 1 hour after the HBC or cyclopamine injections from P22 to P25.

### Immunofluorochemistry

Mice were anesthetized with urethane (1.0 g/kg, i.p.; Cat# U2500, Sigma) and xylazine (13 mg/kg i.p.; Cat# X1251, Sigma). Animals were transcardially perfused with ice-cold phosphate-buffered saline (PBS) followed by 4% PFA for 5–10 min depending on primary antibodies used. The perfusion-fixed mouse brain was postfixed in the same fixative for ~2 hours at 4°C. Free-floating coronal sections (40–50 μm thickness) prepared using a vibratome (Cat# DTK-1000, Dosaka) with the immunohistochemical protocol. For staining with anti-Ki67 antibody, sections were pre-heated in water bath for 30 min in sodium citrate buffer (pH 6.0) at 80°C, cooled to room temperature, and subsequently rinsed in PBS. For anti-BrdU staining, sections were required pre-treatment in 1N HCl for 45 min at 37°C. Following this incubation, sections were neutralized with 0.1 M borate buffer (pH 8.5) for 15 min (twice), then in PBS 5 times for 10 min. For multiple staining using BrdU-stained sections, the sections were incubated in 4% PFA for 1 hr at 4°C before proceeding to the next step. A PBS wash of the sections is followed by an application of a blocking solution (5% normal donkey or goat serum, 0.3% Triton X-100 in PBS) for 3 hrs at room temperature. Primary antibodies (see below) were then incubated overnight at 4°C. After washing in PBS, sections were incubated in secondary antibodies (typically at 1/500; see below) in BS for 1.5 hrs at RT. Cell nuclei were visualized by post-staining with Hoechst 33342 (3 μg/ml; Thermo Fisher). Stained sections were mounted with Vectashield antifade mounting medium (H1000, Vector Lab).

Primary antibodies were rat monoclonal antibodies against BrdU (clone BU1/75(ICR1), GeneTex Cat# GTX26326, RRID:AB_1081056, 1:500; Abcam Cat# ab6326, RRID:AB_305426, 1:500), mouse monoclonal antibodies against Ki67 (BD Biosciences Cat# 556003, RRID:AB_396287, 1:2000), Nestin (clone rat-401, Millipore Cat# MAB353, RRID:AB_94911; DSHB Cat# Rat-401, RRID:AB_2235915, 1:200), CNPase (Millipore Cat# MAB326, RRID:AB_2082608, 1:400), S100β (Sigma-Aldrich Cat# S2532, RRID:AB_477499, 1:1000), GAD67 (Millipore Cat# MAB5406, RRID:AB_2278725, 1:1000), rabbit polyclonal antibodies against Ki67 (Leica Microsystems Cat# NCL-Ki67p, RRID:AB_44210, 1:1000), NG2 (Millipore Cat# AB5320, RRID:AB_91789, 1:400), Olig2 (Millipore Cat# AB15328, RRID:AB_2299035, 1:1000), GFAP (Millipore Cat# AB5804, RRID:AB_2109645, 1:400), Iba1 (Wako, Cat# 019–19741, RRID:AB_839504, 1:500), cleaved Caspase 3 (Cell Signaling Technology Cat# 9661, RRID:AB_2341188, 1:500), and goat polyclonal antibodies against PDGFRα (R&D Systems Cat# AF1062, RRID:AB_2236897, 1:200) and Olig2 (Santa Cruz Biotechnology, sc-19969, RRID:AB_2236477, 1:50).

Secondary antibodies from Jackson ImmunoResearch Laboratories (West Grove, PA) were goat anti-rat IgG conjugated with DyLight405 (112-475-167, RRID:AB_2338314) or AMCA (112-155-167, RRID:AB_2338221), goat anti-rabbit IgG conjugated with Alexa488 (111-545-144, RRID:AB_2338052), goat anti-mouse IgG conjugated Cy3 (115-165-166, RRID:AB_2338692) or goat anti-mouse Fcγ conjugated with Alexa647 (115-605-071, RRID:AB_2338909), and donkey anti-goat conjugated with Alexa555 (ab150130, Abcam, RRID:AB_2715537). Goat anti-rat IgG conjugated with Alexa633 (A21094, Invitrogen, RRID:AB_2535749) was also used when appropriate.

### Imaging and data analysis

Fluorescence images in the binocular region of V1 were captured at 1 μm step using a laser scanning confocal microscope (Leica TCS SP8) with HyD detectors. To define the analysis region in V1, in coronal sections, we first drew a vertical line though the center of the ventral division of the medial geniculate nucleus (MGv), and drew a second line through occipital cortex from the MGv at a 15° angle to the vertical line. This 15° angle line was assumed to run through V1 based on mouse atlas [26]. The regions of interest (ROI) for counting cells was a cortical region delimited by pia, the border between layer 6 and the subcortical white matter, and two parallel lines with each line separated 400 μm medial and lateral from the 15° line. Consecutive five or six sections were used per mouse, and at least three mice were used per group. Optical dissector was used to determine the number of positively labeled cells and analyzed using Fiji/ImageJ software (NIH, USA, RRID:SCR_002285).

Cells with fluorescence intensity above a defined threshold were considered immunopositive cells. For cell counting, the mean background fluorescence intensity was calculated by averaging at least five unstained locations on the same histological sections. Threshold intensity was defined as 3x SD of the mean background intensity in each section and subtracted from the measured intensity in ROI. Cell counting was performed blind to experimental conditions. The cell density (cells/10^6^ μm^3^) was computed by dividing cell counts with laminar volume obtained from 40–50 μm optical sections. ROIs in V1 were divided into 8 bins with layer 1 occupying bin 1 and the remaining ROI being divided into bins 2~8 having an equal cortical depth (12.5% each).

For measuring the pixel-intensity of CNPase (2’,3’-Cyclic-nucleotide 3’-phosphodiesterase)-immunoreactivity, images were exported into NIH image J, and 2 μm thick optical sections were z-stacked and flattened. Threshold was subtracted from the measured intensity as above. The distribution of fluorescence intensity was measured using the profile function of Image J for each section. The mean intensity was then obtained from the average of at least four sections from each animal condition. For cumulative fraction analysis of the intensity, the pixel intensities across the cortical thickness from pia to the white matter were divided into 1% steps, normalized to the total intensity, and cumulative fraction of intensity was analyzed with Kolmogorov–Smirnov test. Data analysis was performed blind to experimental conditions.

The distance between adjacent BrdU+ nuclei was measured in three-dimensional optical sections. BrdU+ cells were considered doublets when the distance between the centers of the nuclei was below 35 μm as described previously [27].

### Statistical analysis

Statistical analyses were performed with Excel (Microsoft) and the R Project for Statistical Computing (RRID:SCR_001905). Statistical significance for two independent variables was examined using unpaired Student’s *t*-test. Two groups of nonparametric values were analyzed using the two-sample Kolmogorov–Smirnov test (K-S test). The variability between group means was compared using one-way or two-way ANOVA with the Tukey-Kramer post hoc test for individual group differences. All statistical tests used the significance level (α) of 0.05. *p* < 0.05 was considered statistically significant. N values represent the number of animals used for each animal group/condition. Data and error bars are reported as mean ± standard error of the mean (S.E.M.).

## Results

### Developmental peak of proliferative cells in V1

**Fig 1:**
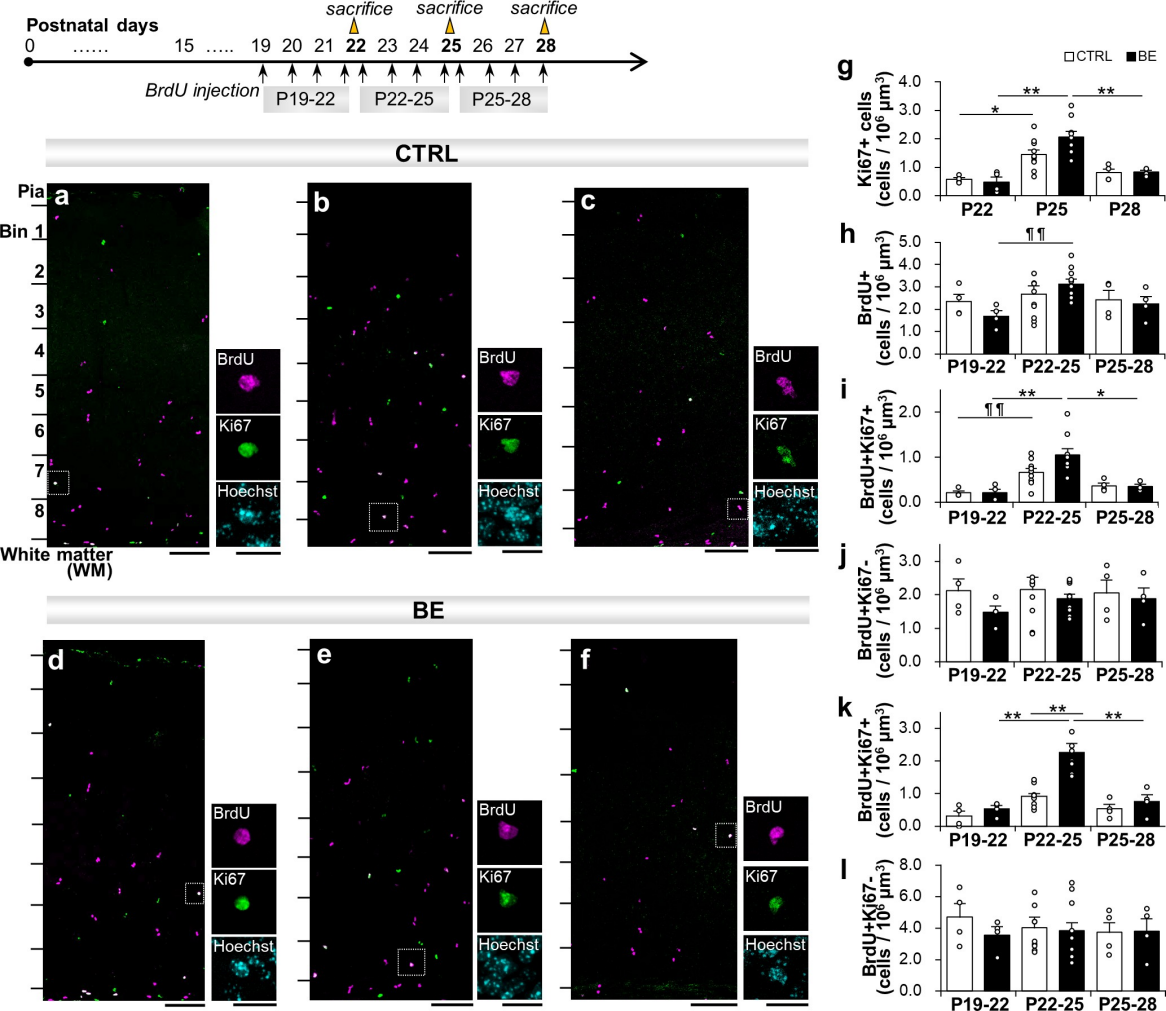
Cell proliferation analysis during postnatal development. Experimental scheme for labeling of proliferated cells. Mice were sacrificed at P22, P25, and P28 following daily injection of BrdU for 3 days (~72 hours). (***a-f*)** Confocal immunofluorescence analysis with Ki67 (green) and BrdU (magenta) staining patterns in V1 in control (***a-c***) and BE mice (***d-f***) at P22 (***a*, *d***), P25 (***b*, *e***), and P28 (***c*, *f***). The magnified images of BrdU+Ki67+ cells are shown on the right side with Hoechst (cyan) staining for the nuclei. Scale bars, 50 μm (left) and 20 μm (right). (***g-j***) Cell densities of Ki67+ (***g***), BrdU+ (***h***), BrdU+Ki67+ (***i***), BrdU+Ki67- (***j***) cells in V1. N = 4 for P22; N = 10 for P25; N = 4 for P28 per group. (***k-l***) The cell density of BrdU+Ki67+ cells (***k***) and BrdU+Ki67- cells (***l***) in the bottom lamina (Bin 8). ¶¶p < 0.01 one-way ANOVA; *p < 0.05; **p < 0.01, Tukey-Kramer post hoc tests after two-way ANOVA.

To assess possible developmental changes in proliferative cells in the binocular region of V1 during the sensitive period for binocular inputs [15], we first monitored the cell density of Ki67-expressing (Ki67+) cells and the proliferative cells by injecting 5-bromo-deoxyuridine (BrdU) over 72 hours with 4 daily injections, starting from P19, P22, and P25, and immunostaining against BrdU and Ki67 (Fig 1). Ki67 is the nuclear protein expressed during the active phase of the cell cycle [28] as well as during a reversible G0/G1 phase before differentiation, where it disappears gradually during quiescence and becomes undetectable in slowly cycling cells in late G1/early S phase [29]. Confocal analyses of the immunofluorescence-labeled cells indicated that Ki67+ cells were sparsely distributed throughout V1 at the three developmental ages (Fig 1). Quantification of Ki67+ cell densities showed a significant increase on P25 in control mice (Fig 1G, one-way ANOVA, F(2, 15) = 7.30, p = 0.0061), where the cell density increased about 2.5-fold from P22 to P25 and tended to decline from P25 to P28 in control mice (Fig 1G). BrdU immunopositive (BrdU+) stains were detected also sparsely in V1 with apparently more distribution toward the lower layers of the cortex. The cell densities of BrdU+ cells were statistically similar at any of the 3-day developmental periods (Fig 1H, one-way ANOVA, F(2, 15) = 0.181, p = 0.84). These data suggest that the proliferation rate of newly proliferating cells is unchanged but the Ki67+ undifferentiated (proliferative) cells are elevated at P25.

Further examination of BrdU+ cells for co-labeling with Ki67 indicated that newly proliferated BrdU+ cells expressing Ki67 were elevated for P22 to P25 (P22-25) in control mice (Fig 1I, one-way ANOVA, F(2, 15) = 7.46, p = 0.0067). Their cell densities were much less than those of the BrdU+ cells that do not express Ki67 (i.e., BrdU+Ki67-; compare Fig 1I and 1J). These results suggest that the 3-day period of P22-25 is a sensitive period for undifferentiation of the proliferated cells in V1. We note that, unlike BrdU+Ki67+ cells, BrdU+Ki67- cell densities were similar for the three age periods (Fig 1J, one-way ANOVA, F(2, 15) = 0.014, p = 0.986), suggesting a continuous supply of cells exiting the cell cycle.

#### Binocular enucleation elevated proliferative cells of P22-25 proliferated cells at the bottom of V1

We then examined the effect of binocular enucleation (BE) on the proliferation and differentiation of Ki67 and BrdU labeled cells in V1. We used mice enucleated both eyes at the time of eye opening (P14-15) and reared until sacrifice. As found in control mice, an average Ki67+ cell density was significantly elevated at P25 in BE mice (Fig 1G; one-way ANOVA, F(2, 15) = 8.33, p = 0.0036). However, there was no significant interaction between visual loss (control and BE mice) and ages (P22, P25, and P28) on Ki67+ cell densities (two-way ANOVA analysis, F(2, 30) = 2.06, p = 0.14), suggesting no difference in proliferative cell formation between control and BE mice over the developmental ages. Meanwhile, an average BrdU+ cell density was elevated at P25 in BE mice (Fig 1H, one-way ANOVA, F(2, 15) = 7.42, p = 0.0058), suggesting that BE affected proliferation rates from P22 to P25. However, there was no statistically significant interaction between visual loss and age on BrdU+ cell densities (two-way ANOVA, F(2, 30) = 1.21, p = 0.31). BE had little effects on BrdU+ cell densities between any three developmental periods (Tukey-Kramer post hoc test, p = 0.093 for P19-22 vs. P22-25, p = 0.54 for P22-25 vs. P25-28). In addition, BE did not significantly increase the cell density over control at any age (Tukey-Kramer post hoc test, p = 0.90 for P22, p = 0.86 for P25, and p = 1.0 for P28). These results suggest that there is developmental influence on proliferative cells to peak at P25 in control and BE mice, but BE had little influence on the number of Ki67+ cells nor BrdU+ cells in V1.

We next examined if BE affected the proliferative state of newly proliferated cells. An average BrdU+Ki67+ cell density for P22-25 was the highest among the three developmental ages in BE mice (Fig 1I, one-way ANOVA, F(2, 15) = 11.1, p = 0.0013). Visual loss and age on BrdU+Ki67+ cell densities over the developmental periods, however, showed no significant interaction (two-way ANOVA, F(2, 30) = 2.09, p = 0.14). An average BrdU+Ki67+ cell density was about 5 times higher for P22-25 than P19-22 and over 2 times higher for P22-25 than P25-28 in BE mice (Fig 1I; Tukey-Kramer post hoc test, p = 0.0034 for P19-22 vs. P22-25, p = 0.015 for P22-25 vs. P25-28). This elevated cell density for P22-25 in BE mice, however, was not significantly different from the BrdU+Ki67+ cell density for control mice (Tukey-Kramer test, p = 0.054). In the meantime, BE did not affect BrdU+Ki67- cell densities for the three developmental ages in BE mice (Fig 1J, one-way ANOVA, F(2, 15) = 1.17, p = 0.34). No interaction of visual loss and age on their cell densities was found (two-way ANOVA, F(2, 30) = 0.33, p = 0.72). And, no statistical significance was detected for control versus BE at any age (Tukey-Kramer post hoc test, p = 0.84 for P19-22, p = 0.97 for P22-25, p = 1.0 for P25-28) nor among any age periods in BE mice (Tukey-Kramer post hoc test, p = 0.094 for P19-22 vs. P22-25, p = 1.00 for P22-25 vs. P25-28). These data indicate that BE elevated proliferative cells at P25, including those newly proliferated cells between P22 to P25, but BE had little influence on the proliferated cells that existed the cell cycle (i.e., BrdU+Ki67- cells).

We then quantified the proliferated cell densities in the lower part of the cerebral cortex, since myelin basic protein (MBP), a mature OL marker, is highly expressed during this developmental period [30] and oligodendrocyte genes are enriched there [18]. V1 was divided into 8 laminas (bins) with layer 1 being bin 1 and the rest of the cortical layers from the layer 1/layer 2 border to the layer 6/subcortical white matter border being divided into 7 bins of equal proportions, which roughly correspond to layer 2, 3, 4, upper layer 5, lower layer 5, upper layer 6, and lower layer 6. Analyses for BrdU+Ki67+ cell densities for the three developmental periods showed that the proliferative cells had significant difference between ages at the bottom lamina (bin 8) (two-way ANOVA test, F(2, 29) = 30.14, p = 8.3x10^-8^). The proliferating cells increased more than 2.5-fold from P22 to P25 and decreased to a similar extent from P25 to P28 in BE mice (Fig 1K, Tukey-Kramer post hoc test, p = 1.1x10^-6^ for P22 vs. P25; p = 2.0x10^-5^ for P25 vs. P28). This elevated level of BrdU+Ki67+ cell density under BE condition was significantly different from the control condition (Fig 1K; two-way ANOVA test, F(2, 29) = 6.97, p = 0.0034; Tukey-Kramer post hoc test, p = 1.9x10^-6^ for control vs BE mice). BrdU+Ki67- cells had no significant differences between ages (Fig 1L; two-way ANOVA test, F(2, 29) = 0.23, p = 0.80; Tukey-Kramer post hoc test, p = 0.78 for P22 vs. P25, p = 0.99 for P25 vs. P28). These results suggest that BE influences the cell state of proliferated cells toward undifferentiation around P25 at least at the bottom lamina of V1, prompting further investigation on properties of proliferated cells.

#### Proliferated cells were oligodendrocyte lineage cells

**Fig 2:**
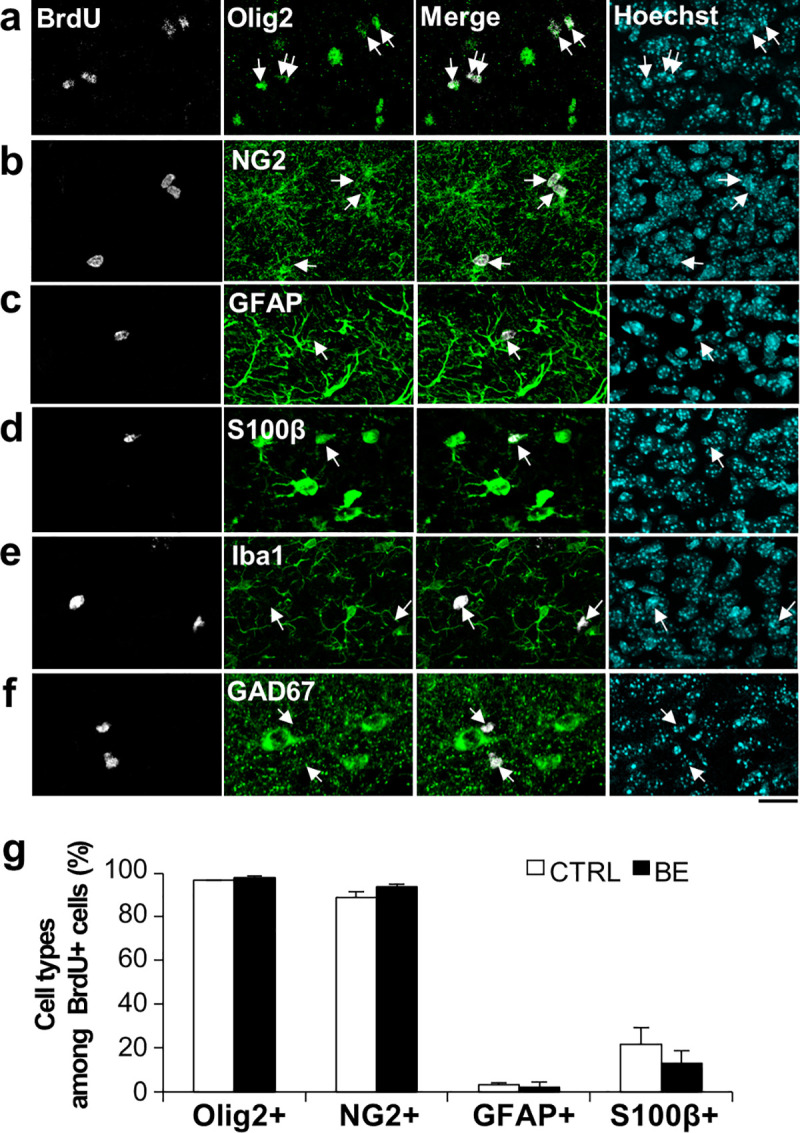
Cell type determination of BrdU+ cells at P25. **(*a-f***) Confocal images of Olig2+ (***a***), NG2+ (***b***), GFAP+ (***c***), S100β+ (***d***), Iba1+ (***e***), or GAD67+ (***f***) cells (green) along with BrdU+ cells (gray, arrows) in the bottom lamina (Bin 8) of V1 at P25. Scale bar, 20 μm. (***g)*** Percentages of Olig2+, NG2+, GFAP+, and S100β+ cells among BrdU-labeled cells. N = 6 for Olig2+; N = 5 for NG2+; N = 3 for GFAP+; N = 5 for S100β+ images. Unpaired Student’s t-test, p > 0.05.

We next examined the cell types of the P22-25 proliferated cells at the peak of undifferentiation (Fig 2). Essentially all BrdU+ cells were oligodendrocyte lineage cells, as about 98% of them expressed Olig2, the transcription factor and nuclear marker for oligodendrocyte lineage cells (Fig 2G). NG2 proteins were expressed in nearly 90% of BrdU+ cells at this age, suggesting that the proliferated cells were mostly OPCs or late progenitors/ pre-oligodendrocytes (pre-OL), which are here defined as NG2+ differentiated OPC lineage cells [31–33]. The remaining 10% of BrdU+ cells lacked NG2 expression and therefore could be glial progenitors or maturing oligodendrocytes. As Olig2-expressing cells could also generate other lineage cell types such as astrocytes [3, 34–39], BrdU+ cells were co-stained against astrocyte markers GFAP or S100β (Fig 2C, 2D and 2G). While GFAP-expressing BrdU+ cells were few, about 20% of them expressed S100β. This relatively high proportion of S100β expression is not surprising since it could be expressed in OPCs [40] and maturing oligodendrocytes. No BrdU+ cells expressed Iba1 (Fig 2E), suggesting that the proliferated cells do not likely include microglia. BrdU+ cells did not express GAD67, a GABAergic neuron marker (Fig 2F). Taken together, these data suggest that the proliferative cells elevated at P25 were essentially all oligodendrocyte lineage cells and mostly NG2 glia.

Additionally, we also examined if BE affected cell densities of these cell types (Fig 2G). We found no evidence of BE-induced changes in them. Since BE altered Ki67 expression without altering BrdU+ cell densities, these data suggest that BE affects cell cycle state or differentiation state without altering cell types.

#### BE increases symmetrically-divided, proliferative cells at the bottom of V1 at P25

**Fig 3:**
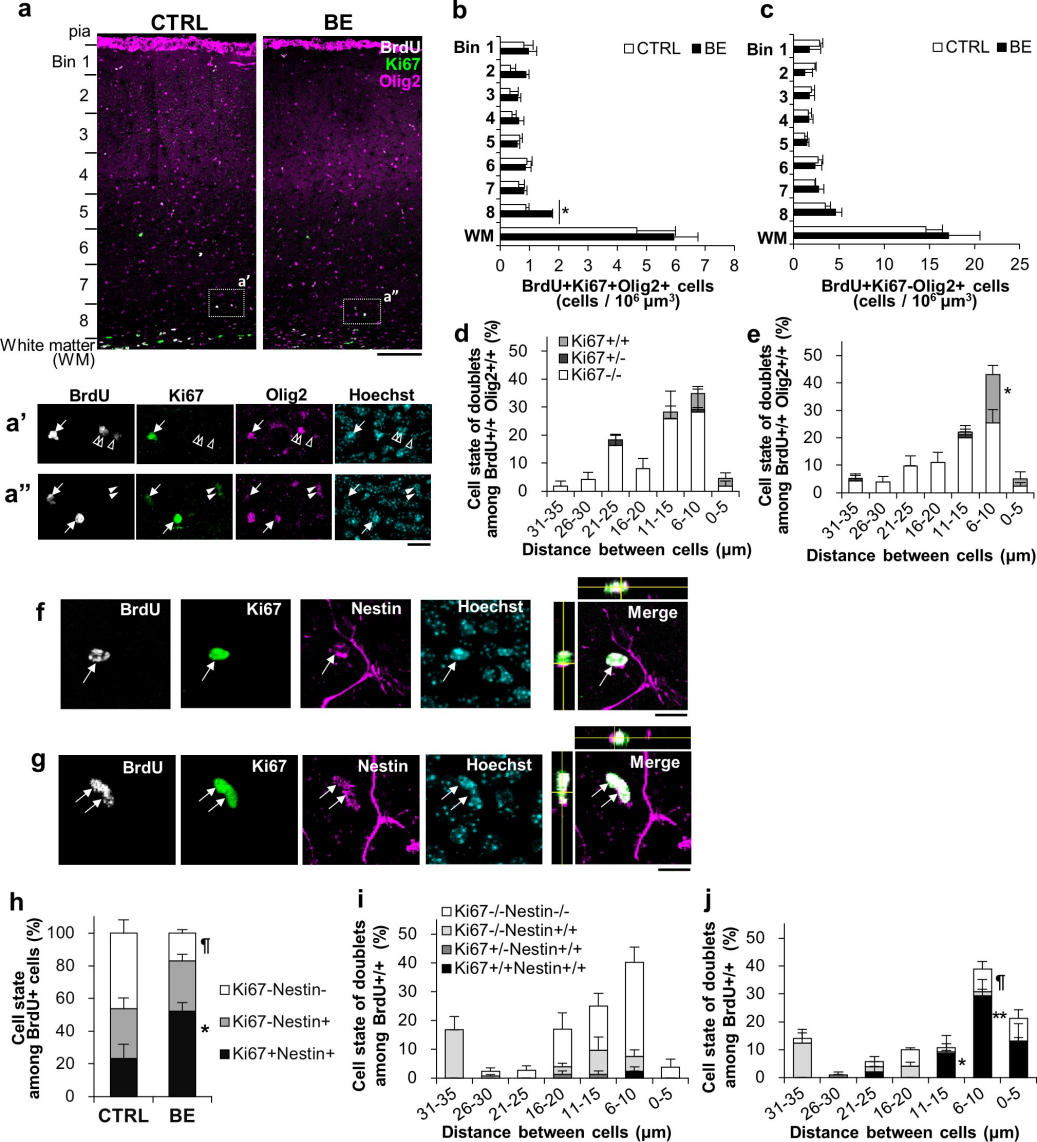
Cell state and cell division symmetry at P25. **(*a-c)*** Laminar analysis of BrdU+ (gray), Ki67+ (green), and Olig2+ (magenta) cells in V1. Boxed areas were magnified below (***a’*** and ***a”***), showing BrdU+Ki67+Olig2+ cells (arrow), BrdU+Ki67-Olig2+ cells (open arrowhead), and BrdU-Ki67+Olig2+ cells (closed arrowhead). Scale bars, 100 μm (***a***), 20 μm (***a’***, ***a”***). The laminar cell densities of Ki67+ proliferative state (***b***) and Ki67- (***c***) OL lineage cells. N = 5 per group, one-way ANOVA, F(1, 8) = 51.91, *p = 0.000092. (***d*, *e***) Cell state analysis of BrdU+Olig2+ sister cells in bin 8 based on Ki67 expression. Symmetrically proliferative cells (Ki67+/+), proliferative and differentiated asymmetric doublets (Ki67+/-), and symmetrically differentiated cells (Ki67-/-) were compared between control (***d***) and BE (***e***) mice at each distance range using one-way ANOVA with Tukey-Kramer post hoc test: F(1, 8) = 7.94, *p = 0.023, N = 5 for each mouse group. (***f-j***) Cell state analysis of BrdU+ (gray) proliferated cells using Ki67 (green) and Nestin (magenta) in bin 8 of control (***f***) and BE (***g***) mice. Ki67+Nestin+ (arrow) among BrdU+ cells. The x-z and y-z cross-section views of white box images are shown at the top and left of the box, respectively. Scale bars, 20 μm. (***h*)** Proportion of BrdU+ singlet cells expressing Ki67 and/or Nestin. N = 5 for each mouse group, one-way ANOVA with Tukey-Kramer post hoc test, F(1, 8) = 8.31, *p = 0.020 for Ki67+Nestin+, F(1, 8) = 12.41, ¶p = 0.0078 for Ki67-Nestin-. (***i*, *j***) Cell state analysis of BrdU+ doublets for Ki67 and/or Nestin expression in control (***i***) and BE mice (***j***). N = 5 per group, one-way ANOVA, F(1, 8) = 17.04, ¶p = 0.0033 for Ki67-/-Nestin-/-, F(1, 8) = 9.43, *p = 0.015, F(1, 8) = 20.29, **p = 0.0020 for Ki67+/+Nestin+/+.

Given that P22-25 was found to be a developmentally unique period for proliferative cell populations in V1, we decided to further explore the nature of the BrdU+ proliferated cells and the effects of BE on their proliferation and/or differentiation. First, the P22-25 BrdU labeled cells were co-stained against Ki67 as well as Olig2 and analyzed for possible laminar differences in their cell states (Fig 3). BrdU+Olig2+Ki67+ cells were generally found more in the lower laminas (bins 6~8) than in the upper middle laminas (bins 2~5) of the cortex. In any bins, BrdU+Olig2+ cells were more Ki67- than Ki67+ as found for BrdU+ cells throughout the entire laminas (Fig 1I and 1J).

To reveal possible laminar dependence of BE-induced changes in the proliferation and differentiation of OPCs, we analyzed the cell densities of BrdU+Olig2+ cells in BE mice. As detected above, the bottom lamina (bin 8) was responsive to BE, where the cell density of BrdU+Olig2+Ki67+ cells almost doubled (Fig 3B, one-way ANOVA with Tukey-Kramer post hoc test, F(1, 8) = 51.91, *p* = 0.000092), while BrdU+Olig2+Ki67- cell density was unaffected by the visual deprivation (Fig 3C). These results confirm that the bottom lamina, a part of layer 6 in V1, is sensitive to BE, where a proliferative state of the proliferated cells is greatly elevated without significant changes in their differentiation.

To further characterize the BE-induced cell state changes, we next examined the symmetry of cell division in the BrdU+Olig2+ cells (Fig 3D and 3E). Previous studies in postnatal cortexes identified proliferated OPCs as pairs of “sister cells” based on their proximal distribution [14, 27, 41, 42]. We found that over half of BrdU-labeled nuclei existed as “sister cells” or doublets (Fig 3A’–3A”). In control mice, most BrdU+ doublets were distributed closely within 15 μm from each other, and the proportion of the pairs declined as the distance increased toward 35 μm. The majority of postmitotic doublets in bin 8 was Ki67-/- doublets, and Ki67+/+ doublets were less (Fig 3D). Asymmetrically-divided (or dividing) cells (Ki67+/-, one expressing Ki67, the other not) were rare or none in control mice. In BE mice, as were in control mice, the most populated BrdU+ doublets were Ki67-/-. BE, however, affected BrdU+ cells to become Ki67+ proliferative cells, where there was about 3-fold increase in the symmetrically-divided Ki67+ doublets present within 15 μm (Fig 3E; for 6~10 μm, 5.6 ± 2.3% for control and 17.1 ± 3.2% for BE; one-way ANOVA, F(1, 8) = 7.94, *p* = 0.023). Asymmetrically-divided cells were rare in BE mice as well. These data suggest that BE increases proliferative BrdU+ cells by inducing symmetrical cell division of the proliferated cells.

#### BE increased proliferative OPCs by decreasing maturing OLs without affecting quiescent cells at P25

Since the source of the BE-elevated proliferative cells is unclear, we next examined cell state changes among P22-25 proliferated cells using a combination of Ki67 and Nestin staining. Nestin is an undifferentiated cell marker expressed in proliferative glial progenitor cells (GPCs) and OPCs, but not in differentiated oligodendrocytes (premyelinating/immature OLs or myelinating/mature OLs [43], *reviewed in* [44–46]). Since Ki67 is present during the cell cycle and degraded during the G0 phase toward differentiation [29], we presume that co-expression of Ki67 and Nestin (Ki67+Nestin+) indicates a cell cycling or reversible G0/G1 (proliferative and undifferentiated) state (GPCs or OPCs), the absence of both Ki67 and Nestin (Ki67-Nestin-) indicates an OL-committed, differentiated maturing state (pre-OLs or mature OLs), and an expression of Nestin but not Ki67 (Ki67-Nestin+) indicates a potentially quiescent undifferentiated state. With these assumptions, about 20% of BrdU+ cells were in the proliferative undifferentiated state, about 30% of BrdU+ cells were in the quiescent undifferentiated state, and about 50% was in the maturing differentiated state (Fig 3F–3H). The proportional analysis of BrdU+ cells showed that BE increased the proliferative undifferentiated state about 3-fold over control and decreased the maturing differentiated state about 3-fold without affecting the proportion of the quiescent state (Fig 3H, one-way ANOVA with Tukey-Kramer post hoc test, F(1, 8) = 8.31, *p* = 0.020 for control vs. BE of BrdU+Ki67+Nestin+; F(1, 8) = 12.41, *p* = 0.0078 for control vs. BE of BrdU+Ki67-Nestin-). Since there was no significant difference in the cell densities of BrdU+Ki67- cells between control and BE mice (Control: 4.03 ± 0.52 cells/ 10^6^ μm^3^; BE: 3.84 ± 0.60 cells/ 10^6^ μm^3^; Tukey-Kramer post hoc test, p = 0.95) (see also Figs 1L and 3C), it is likely that BE dynamically shifted the differentiation process toward proliferative undifferentiation by reducing symmetric maturation without affecting quiescent state formation.

The analysis of nascent BrdU+ doublets further supports this conclusion of the cell state shift toward undifferentiation in the proliferated cells. In control mice, symmetrically divided Ki67-/-Nestin-/- (differentiated) doublets were the dominant cell types with a majority separating 6–20 μm from each other (Fig 3I). BE resulted in a clear increase over control in symmetrically-divided proliferative undifferentiated cells (Ki67+/+Nestin+/+) that are closely distributed within 15 μm of each other (one-way ANOVA, F(1,8) = 20.3, p = 0.0020 for 6–10 μm) and a decline of differentiated maturing cells (one-way ANOVA, F(1, 8) = 17.04, p = 0.0033 for 6–10 μm, F(1, 8) = 9.43, p = 0.015 for 11–15 μm)(Fig 3I and 3J). Most symmetrically-divided, presumptive quiescent doublets (Ki67-/-Nestin+/+) were segregated distantly from each other, and those that are present near each other were much fewer in proportion. Their proportions were similar between control and BE mice, consistent with no change in the quiescent singlet progenitor density. A very small proportion of asymmetrically-divided proliferative and quiescent doublets were also found in both control and BE mice, possibly implicating a slight temporal difference in the divided (or dividing) doublets. Overall, along with singlet analysis, we conclude that BE promotes the nascent BrdU+ cells to stay proliferative (undifferentiated), delaying the normal transition to maturation (differentiation), without much affecting the transition to quiescence.

#### BE delays lineage progression toward differentiation without influencing cell division symmetry

**Fig 4:**
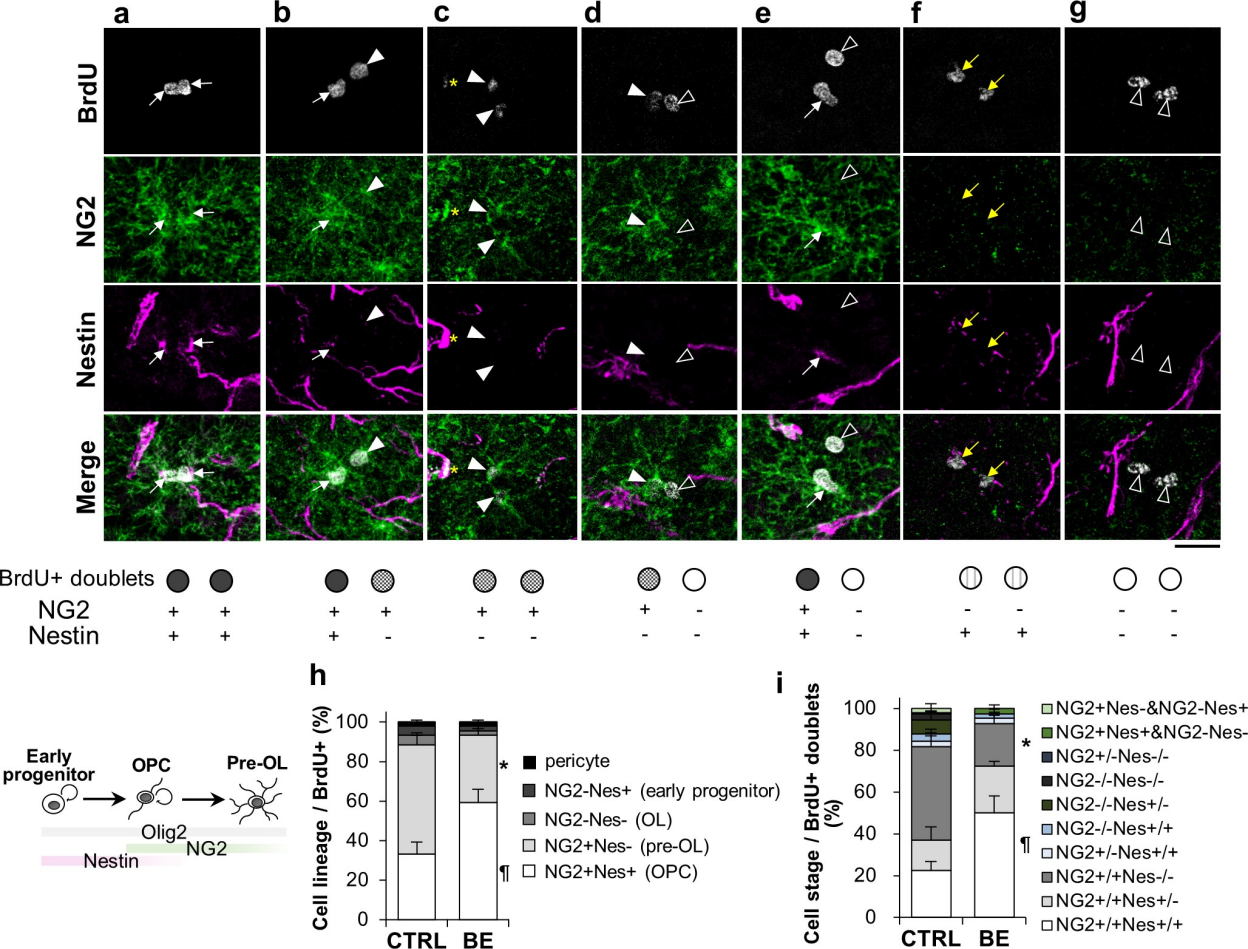
Cell stage progression of proliferated cells at P25. **(*a-g***) Confocal images of BrdU+ (gray) sister cells co-immunostained with antibodies against NG2 (green) and Nestin (magenta) in bin 8. Shown are representative images of BrdU+ doublets: NG2+/+Nestin+/+ (***a***), NG2+/+Nestin+/- (***b***), NG2+/+Nestin-/- (***c***), NG2+/-Nestin-/- (***d***), NG2+Nestin+ & NG2-Nestin- (***e***), NG2-/-Nestin+/+ (***f***), and NG2-/-Nestin-/- (***g***). Arrows and arrowheads indicate as follows: NG2+Nestin+ (white arrow), NG2+Nestin- (closed white arrowhead), NG2-Nestin- (open white arrowhead), and NG2-Nestin+ (yellow arrow). Yellow asterisks indicate NG2 and Nestin-colabeled pericytes. Scale bar, 20 μm. Schematic diagram is given on the left to show the definition of putative OL lineage cell types based on possible expression patterns during cell stage progression. (***h***) The proportion of NG2 and/or Nestin expressing singlet cell types among BrdU-labeled cells. Presumptive cell types are indicated in the parentheses. For CTRL vs. BE, one-way ANOVA and Tukey-Kramer post hoc test, F(1, 8) = 7.89, *p = 0.023, F(1, 8) = 7.49, ¶p = 0.026. (***i***) Proportional analysis of BrdU+ doublets. For CTRL vs. BE, N = 5 per group, one-way ANOVA and Tukey-Kramer post hoc test, F(1, 8) = 8.28, *p = 0.021, F(1, 8) = 9.28, ¶p = 0.016.

Our data so far demonstrated that nearly half of the P22-25 BrdU+ cells were differentiated at P25, and BE decreased maturing cells while increasing proliferative OPCs. These data predict that BE would decrease the formation of pre-OLs and increase OPCs. To test this, BrdU+ cells were co-immunostained against NG2 and Nestin, and the proportion of BrdU+ cells expressing NG2 and/or Nestin was measured. NG2 is expressed mainly in OPCs and progressively degraded during differentiation, while Nestin is expressed in both GPCs and OPCs but not in maturing OLs. Therefore, we interpreted that NG2-Nestin+ cells, NG2+Nestin+ cells, NG2+Nestin- cells, and NG2-Nestin- cells would represent GPCs, OPCs, pre-OLs, and OLs, respectively. In control mice, most BrdU+ cells were OPCs (33.2 ± 6.2%) and pre-OLs (55.2 ± 5.1%), while other cell types were small in proportion (Fig 4A–4H). BE significantly increased the proportion of OPCs (59.2 ± 6.9%) and decreased pre-OLs (34.2 ± 5.8%) (Fig 4H; one-way ANOVA with Tukey-Kramer post hoc test, F(1, 8) = 7.89, *p* = 0.023 for NG2+Nestin+ OPCs, F(1, 8) = 7.49, *p* = 0.026 for NG2+Nestin- pre-OLs for control vs. BE), but it did not alter the proportions of other cell types. These data suggest that BE prevented the progression from OPCs to pre-OLs, consistent with the idea that the visual deprivation shifted the OL-lineage cells toward proliferative undifferentiation.

Adult OPCs frequently divide asymmetrically to generate an NG2-expressing proliferative progeny and an NG2-lacking differentiating OL progeny [12, 27]. To determine whether BE affects the symmetry of the differentiating OL lineage cells, we applied the doublet analysis. The largest population of BrdU+ sister cells was symmetrically-differentiated pre-OL (44.8 ± 6.5%) in control mice (Fig 4I). BE downregulated this proportion about 2-fold (20.4 ± 5.4%). This downregulation was associated with approximately 2-fold upregulation of symmetrically-divided OPCs (22.6 ± 4.2% for control, 50.1 ± 8.0% for BE). There were little effects of BE on other doublet cell types, including those that asymmetrically-divided. Taken together, along with the results above, these data suggest that BE promotes the delay in OL lineage progression and an increase in proliferative OPCs without altering the symmetric mode of cell division.

#### Sonic hedgehog signaling may participate in BE-induced proliferative undifferentiation

**Fig 5:**
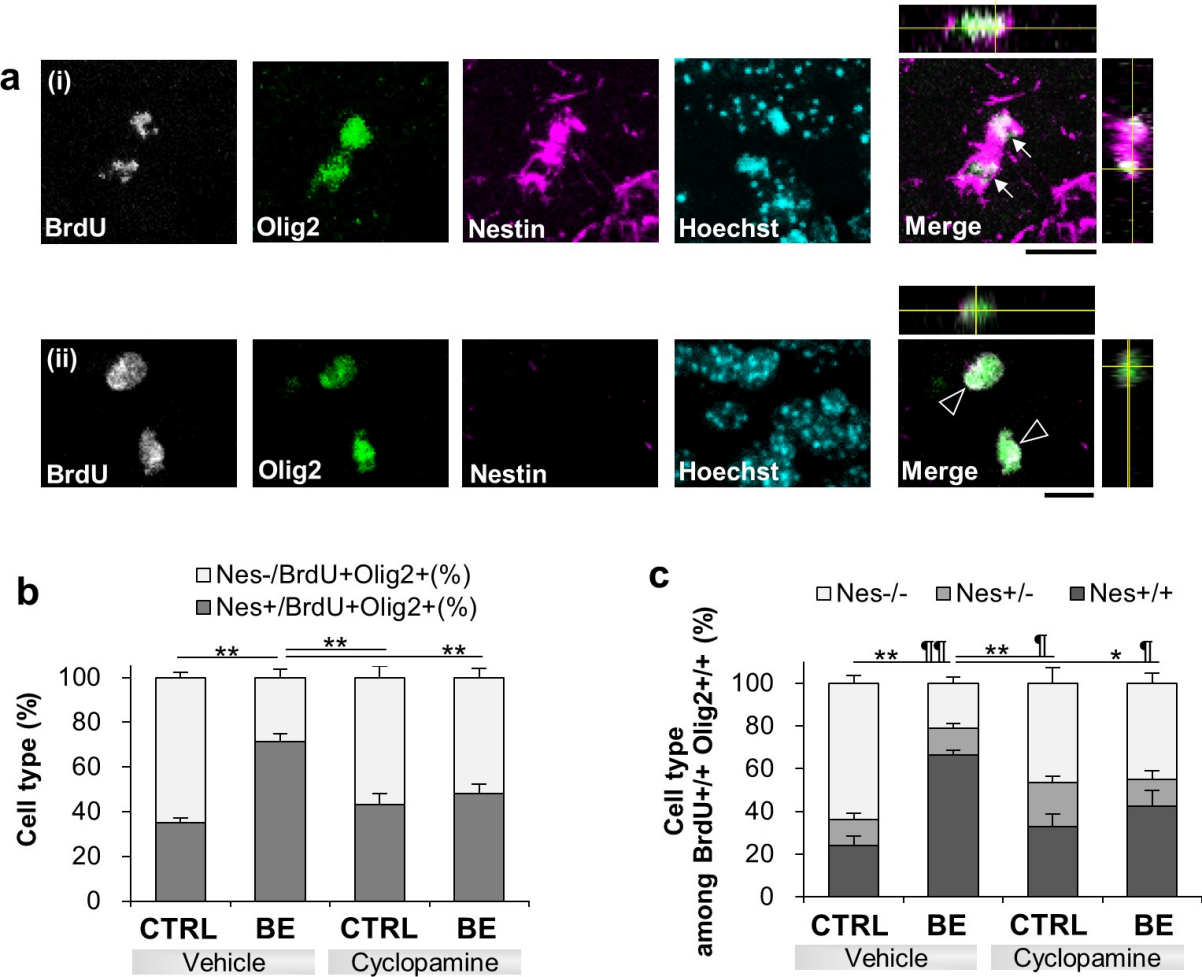
The role of sonic hedgehog signaling in BE-induced undifferentiation at P25. **(*a***) Confocal images showing immunostaining against BrdU (gray), Olig2 (green), Nestin (Nes, magenta) along with Hoechst for vehicle-injected control mice. A merged image of the top row omitted Hoechst for clarity. The x-z and y-z cross-section views of the merged image are shown at the top and right side of the image, respectively. White arrows (a_i_) and open arrowheads (a_ii_) indicate BrdU+Olig2+Nestin+ and BrdU+Olig2+Nestin- cells, respectively. Scale bar, 50 μm. (***b***, ***c***) Percentages of Nestin labeling among BrdU+Olig2+ singlet cells (***b***) and doublet cells (***c***) for the four conditions described above. *p < 0.05; **p < 0.01 for Nestin+ or Nestin+/+, and ¶p < 0.05; ¶¶p < 0.01 for Nestin-/-, two-way ANOVA and Tukey-Kramer post hoc test, N = 5 per condition.

We next sought a mechanism underlying the BE-induced increase in proliferative undifferentiation of OPCs. One possible molecule that controls the proliferation of OPCs would be sonic hedgehog (Shh), whose signaling promotes the generation of OPCs in dorsal ventricular subventricular zone for their progeny to populate in the corpus callosum [47, 48]. To examine if Shh signaling contributed to the BE-induced symmetrically-divided proliferative undifferentiation in the bottom lamina at P25, we systemically injected cyclopamine (25 mg/kg, i.p.), a Smo-binding Shh signaling blocker, for 3 days along with BrdU injection in vivo. BE increased the proportion of Nestin+ cells among BrdU+Olig2+ cells as expected for retarding the progression of OL differentiation (Fig 5A and 5B; two-way ANOVA test, F(1, 16) = 16.67, p = 0.00087; Tukey-Kramer post hoc test, p = 0.000029 for vehicle in control vs. vehicle in BE). This increase was inhibited by cyclopamine (Tukey-Kramer post hoc tests, p = 0.00048 for vehicle in BE vs. cyclopamine in control, *p* = 0.0029 for vehicle in BE vs. cyclopamine in BE), suggesting a role of Shh signaling in BE-induced proliferative undifferentiation.

We also assessed if cyclopamine could block the BE-induced increase in symmetrically-segregated undifferentiation of the proliferated cells. BE elevated the proportion of symmetrically undifferentiated Nestin+/+ cells among BrdU+Olig2+ doublets, and this increase was blocked by cyclopamine (Fig 5C; two-way ANOVA test, F(1, 16) = 9.28, p = 0.0077 for interaction of drug (vehicle, cyclopamine) and mice (control, BE), Tukey-Kramer post hoc tests: p = 0.00024 for vehicle injection in control vs. BE mice, p = 0.0025 for vehicle in BE vs. cyclopamine in control, p = 0.030 for vehicle vs. cyclopamine in BE mice). While keeping the proportion of asymmetrically divided Nestin+/- cells unchanged (two-way ANOVA, F(1, 16) = 0.199 for drug and mice interaction), BE reduced the proportion of the symmetrically differentiated Nestin-/- cells, which was also blocked by cyclopamine (two-way ANOVA test, F(1, 16) = 17.25, p = 0.00075 for drug and mice interaction, Tukey-Kramer post hoc tests: p = 0.000086 for vehicle in control vs. BE, p = 0.011 for vehicle in BE vs. cyclopamine in control, and p = 0.017 for vehicle vs. cyclopamine in BE). These results suggest that Shh signaling is involved in the BE-induced symmetric shift toward undifferentiation.

#### The lineage progression of P22-25 proliferated OPCs beyond P25

**Fig 6:**
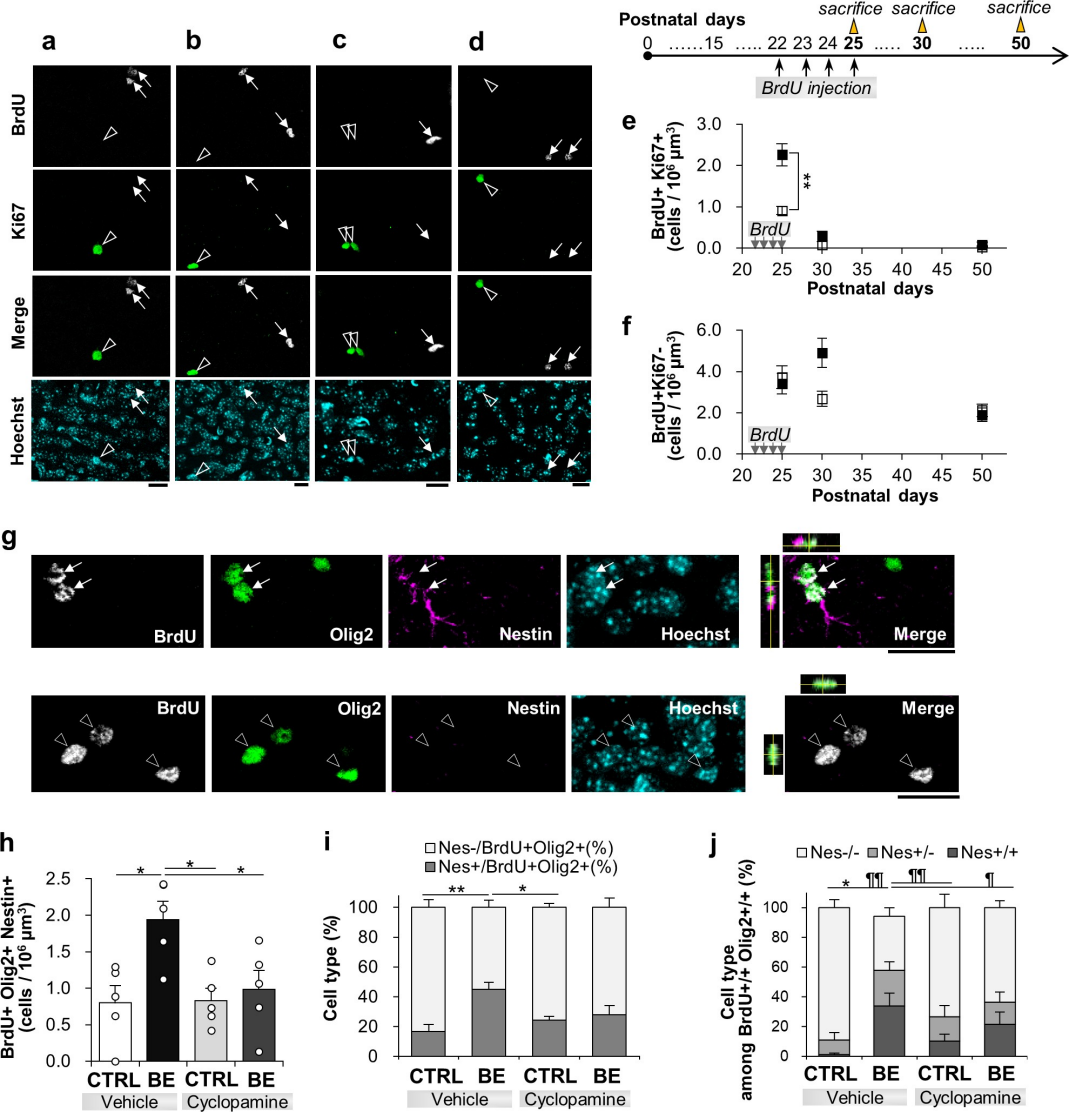
Lineage progression of P22-25 proliferated OPCs. **(*a-d***) Confocal images immunostained against BrdU+ (gray) and Ki67+ (green) and Hoechst counter stain (cyan) in bin 8 at P30 (***a*, *b***) and P50 (***c*, *d***) for control (***a***, ***c***) and BE (***b***, ***d***) mice. Merged images omitted Hoechst for clarity. Scale bars, 10 μm. Scheme on the right shows the experimental protocol for BrdU daily injection from P22 to P25 (arrows) and the dates of sacrifice at P25, P30, and P50 (triangles). (***e*, *f***) Quantification of the cell density of (***e***) BrdU+Ki67+ and (***f***) BrdU+Ki67- cells. **p < 0.01, Tukey-Kramer post hoc tests following two-way ANOVA, N = 10 for P25, N = 4 for P30, N = 5 for P50. (***g***) Confocal images for BrdU (gray), Olig2 (green), Nestin (magenta) co-labeled with Hoechst at P30. Merged images omitted Hoechst for clarity. White arrows and open arrowheads indicate BrdU+Olig2+Nestin+ and BrdU+Olig2+Nestin- cells, respectively. The x-z and y-z cross-section views of white box images are shown at the top and right side of the box, respectively. Scale bar, 20 μm. (***h***) The cell density analysis of BrdU+Olig2+Nestin+ cells for four different conditions. (***i***, ***j***) Percentages of Nestin labeling among BrdU+Olig2+ singlet cells (***i***) and doublets (***j***) for the four conditions described above. *p < 0.05, **p < 0.01 for Nestin+ or Nestin+/+, and ¶p < 0.05, ¶¶p < 0.01 for Nestin-/-, two-way ANOVA with Tukey-Kramer post hoc test, N = 5 per condition.

Next, we examined the lineage progression of P22-25 BrdU-labeled cells. First, we examined the cell state of P22-25 proliferated cells at P30 (Fig 6). In control mice, BrdU+Ki67+ cells were mostly absent (Fig 6A and 6E), indicating that the BrdU+ cells exited the cell cycle and mostly differentiated or became quiescent within 5 days of BrdU secession. In the meantime, BrdU+Olig2+Nestin+ cell density appeared to decline, but not significantly, from P25 (1.77 ± 0.36 cells/10^6^ μm^3^, N = 5) to P30 (0.80 ± 0.23 cells/10^6^ μm^3^, N = 5) in control mice (Fig 6H; one-way ANOVA test, F(1.8) = 5.07, p = 0.054). The cell densities of BrdU+Olig2+Nestin- cells were also unchanged from P25 (3.23 ± 0.65 cells/10^6^ μm^3^) to P30 (3.57 ± 0.64 cells/10^6^ μm^3^) (one-way ANOVA test, F(1,8) = 0.14, p = 0.72). Thus, undifferentiated and differentiating OPCs remained mostly unchanged over the 5 days. Together with the analysis of Ki67 expression, these results suggest that the proliferative cells at P25 exited the cell cycle and became mostly quiescent (Nestin+Ki67-), rather than differentiated (Nestin-Ki67-), at P30 in control mice.

#### BE is unlikely to affect the cell state transition toward quiescence from P25 to P30

Next, BE effects on the lineage progression of P22-25 proliferated OPCs were assessed. The cell density of BrdU+Ki67+ cells was elevated about 2-fold in BE mice compared to control mice at P25 (Fig 6E) as observed above. After 5 days at P30, the cell density was approximately 82.8 ± 8.0% less than that at P25. The extent of this decrease of the proliferative cells was similar to that of control mice (91.3 ± 8.7%). Given that BrdU+ cell densities for control and BE mice were similar at each age (see above for P25; control: 4.47 ± 0.80 cells/10^6^ μm^3^, BE: 4.55 ± 0.73 cells/10^6^ μm^3^ for P30), the loss of Ki67 expression in similar proportions raises the possibility that BE had little influence on this cell state transition to quiescence.

Further support for the lack of BE effects on the transition came from the study of Shh signaling. We examined the transition of P22-25 BrdU+ cells from P25 to P30 by continuously treating mice with cyclopamine or its vehicle beyond final BrdU injection at P25 until the day before sacrifice at P30. At P30, a two-way ANOVA test of the effect of the drug exposure and the visual loss on the cell densities of BrdU+Olig2+Nestin+ cells indicated a statistically significant interaction (Fig 6H; F(1, 16) = 4.59, *p* = 0.048). BE increased their cell density over control, while cyclopamine injection prevented it (Tukey-Kramer post hoc test, p = 0.014 for vehicle injection in control vs. BE, p = 0.043 for vehicle vs. cyclopamine in BE, p = 0.017 for vehicle in BE vs. cyclopamine in control, p = 1.00 for vehicle vs. cyclopamine in control). The cell densities of BrdU+Olig2+Nestin+ cells at P25 were 1.77 ± 0.36 cells/ 10^6^ μm^3^ and 4.48 ± 0.43 cells/ 10^6^ μm^3^ for control and BE mice, respectively (one-way ANOVA, F(1, 8) = 22.96, p = 0.0014). Since their cell densities at P30 were 0.80 ± 0.23 cells/ 10^6^ μm^3^ and 1.94 ± 0.25 cells/ 10^6^ μm^3^ for control and BE mice (Fig 6H), respectively, the percentage decline of cell densities from P25 to P30 was 46.3 ± 24.3% for control and 55.5 ± 6.7% for BE mice (one-way ANOVA, F(1, 8) = 0.133, p = 0.72). Thus, despite the BE effect on the cell densities at each age, the proportional decline in Nestin expression was similar between control and BE mice. A two-way ANOVA analysis of the effect of 5-day aging and the visual loss on BrdU+Olig2+Nestin+ cell densities indicate no interaction (F(1, 32) = 1.24, p = 0.27), suggesting little influence of BE on the likely transition to quiescence.

#### BE affects the symmetric shift to undifferentiation via Shh signaling

We then examined a possible role of Shh signaling in the symmetric mode of cell division toward differentiation at P30. At this age, the majority of BrdU+/+Olig2+/+ doublets lacked Nestin expression (i.e., Nestin-/-) in vehicle-injected control mice (88.9 ± 5.5%; Fig 6J), suggesting that recently divided cells were mostly differentiated. Nestin+/+ doublets were very few with only one of 5 experiments showing Nestin+/+ doublets (0.22 cells/10^6^ μm^3^). In BE mice, Nestin+/+ cells was about one third (34.0 ± 8.5%) of the BrdU+Olig2+ doublets with their cell density being 0.67 ± 0.19 cells/10^6^ μm^3^, and another third of them (36.3 ± 5.8%) was Nestin-/- with their cell density being 0.64 ± 0.08 cells/10^6^ μm^3^. Since the cell densities of BrdU+/+Olig2+/+ doublets were similar between control (1.91 ± 0.67 cells/10^6^ μm^3^) and BE mice (1.89 ± 0.29 cells/10^6^ μm^3^), the proportional differences of Nestin expression would indicate a BE-induced shift in the cell state toward an undifferentiated state of OPCs.

In order to test if Shh signaling could contribute to the shift in undifferentiation, we conducted two-way ANOVA analysis to examine the effect of the visual loss and drug treatment on Nestin expression. The proportion of Nestin-/- doublets was significantly affected by the two factors (F(1, 16) = 10.7, p = 0.0047). BE mice had significantly less Nestin-/- doublets than control mice, and cyclopamine treatment prevented the lowering of Nestin expression (Tukey-Kramer post-hoc tests, p = 0.00017 for vehicle-injected control vs. BE mice; p = 0.042 for vehicle- vs. cyclopamine-injected BE mice; p = 0.70 for cyclopamine-injected control vs. BE mice). BE had little influence on asymmetrically Nestin-expressing OPCs (two-way ANOVA, F(1, 16) = 1.47, p = 0.24). These results suggest that Shh signaling plays a role in the BE-induced shift of symmetrically divided OPCs to become more undifferentiated at P30.

#### OPC differentiation of P22-25 proliferated cells after P30 in control mice

**Fig 7:**
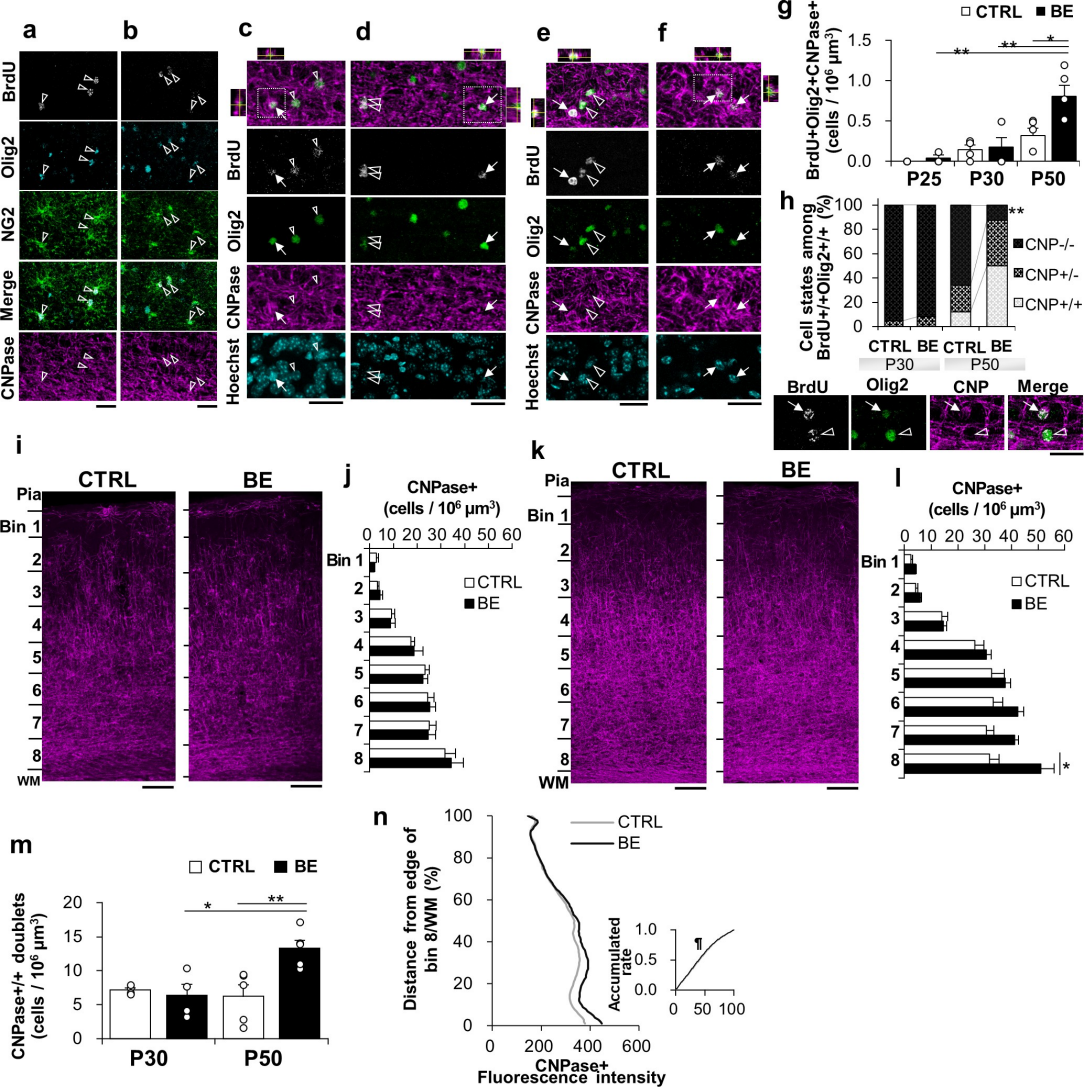
Maturation of P22-25 proliferated OPCs. **(*a*, *b***) Confocal images of BrdU (gray), Olig2 (cyan), NG2 (green), and CNPase (magenta) at P25 for control (***a***) and BE (***b***) mice. Most of the BrdU-labeled cells were Olig2+NG2+CNPase- cells. Merged images of the fourth row omitted CNPase for clarity. Arrowheads indicate BrdU+Olig2+NG2+CNPase- cells. Scale bar, 20 μm. (***c-f***) Confocal images of BrdU (gray), Olig2 (green), and CNPase (magenta) stain with Hoechst (cyan) in bin 8 of V1 at P30 (***c*, *d***) and P50 (***e*, *f***) for control (***c*, *e***) and BE (***d*, *f***) mice. Merged images of the top row omitted Hoechst for clarity. Arrows and open arrowheads indicate maturing oligodendrocyte (BrdU+Olig2+CNPase+) and OPC/pre-OL (BrdU+Olig2+CNPase-), respectively. The x-z and y-z cross-section views of white box images are shown at the top and left/right side of the box, respectively. Scale bars, 20 μm. (***g***) Cell densities of maturing oligodendrocytes (BrdU+Olig2+CNPase+) at P25, P30, and P50. (***h)*** Percentage of CNPase labeling among BrdU+Olig2+ doublets at P30 and P50. (***i-l)*** CNPase (magenta) expression in V1 at P30 (***i***) and P50 (***k***). Scale bars, 100 μm. Laminar analysis of CNPase-labeled cells throughout the cortex at P30 (***j***) and P50 (***l***). *p < 0.05, one-way ANOVA. **(*m***) The cell density of CNPase+ doublets in bin 8 at P30 and P50. For (***g)***, (***h)***, and (***m)***, *p<0.05; **p<0.01, two-way ANOVA with Tukey-Kramer post hoc tests, N = 3 for P25; N = 4 for P30; N = 5 for P50. (***n***) CNPase+ fluorescence intensity in V1 at P50. *Insert*, Cumulative distribution analysis using Kolmogorov–Smirnov test shows more CNPase expression in BE mice: ¶p = 0.00003 (*D-stat* = 0.020, *D-crit* = 0.011).

Having established that P22-25 proliferated cells mostly transitioned to quiescence, we then wondered if they remained quiescent or they differentiated into oligodendrocytes (OL) later in developing control mice. The lineage progression of the proliferated cells was analyzed by staining against CNPase, a marker for maturing OLs (Fig 7). CNPase-expressing (CNPase+) BrdU+ cells were absent at P25 in control mice (an average of 53 ± 5 BrdU+Olig2+ cells examined, N = 3). At P30, however, CNPase+ cells were detected, albeit at a low cell density (Fig 7C and 7G), representing 3.2 ± 1.2% of BrdU+Olig2+ cells (Fig 7H, N = 4). Since the cell density of the BrdU+Ki67- cell cycle-exited OPCs was much greater (Fig 6F, 2.67 ± 0.37 cells/10^6^ μm^3^) than that of BrdU+CNPase+ maturing OLs (Fig 7G, 0.15 ± 0.06 cells/10^6^ μm^3^), and since the majority of BrdU+Olig2+ cells were Nestin- differentiated cells (Fig 6I), many differentiated BrdU+ cells likely exist as CNPase-, presumptive pre-OLs with only a small fraction progressed to mature OLs at P30. Interestingly, we noted that CNPase+ cells detected at P30 formed asymmetrically (Fig 7H). The asymmetric production of the differentiated cells continued at least until P50, increasing about 4-fold in its proportion among BrdU+Olig2+ doublets. At the same time, symmetrically differentiated, maturing OLs also appeared, suggesting an increased demand for maturation for the P22-25 proliferated cells as mice age.

#### BE elevated maturating OLs in the bottom lamina of V1

We next investigated BE effects on the destiny of the proliferated cells. BrdU+Olig2+CNPase+ maturing OLs were present but few at P30, and BE had little influence on their maturation (Fig 7G). While the proportion of CNPase+ cells among BrdU+Olig2+ cells apparently increased about 2-fold from P30 to P50 in control mice, their cell density increased more than 4-fold in BE mice (two-way ANOVA test, F(2, 18) = 3.54, p = 0.050, Tukey-Kramer post hoc test, p = 0.0026 for P30 vs P50 BE mice, p = 0.79 for P30 vs P50 control mice). As a result, the cell density of the BrdU+ maturing OLs was about 2.5-fold higher for BE mice than for control mice at P50 (Tukey-Kramer test, p = 0.016 for control vs BE mice at P50). This increase involved symmetrically and asymmetrically segregated sister cells that were distributed closely within 35 μm (Fig 7C–7F and 7H). Expanding this analysis to the rest of the cortex revealed that CNPase+ cells (both BrdU-labeled and non-labeled) were elevated only in the deep lamina (bin 8) of BE mice (Fig 7K, 7l; one-way ANOVA test, F(1, 8) = 6.88, p = 0.031). CNPase+ doublets were also significantly increased at P50 (Fig 7M; two-way ANOVA test, F(1, 14) = 8.02, p = 0.013, Tukey-Kramer post hoc test, p = 0.0092 for control vs BE mice at P50). This finding corroborated with the elevated intensity of CNPase immunopositive stains for BE mice in the lower part of V1 (Fig 7N; K-S test, p = 0.00003). Taken together, these data indicate that BE upregulated CNPase+ maturing OLs, recruiting the BE-induced P22-25 BrdU+ undifferentiated OPCs, in the bottom lamina of V1.

## Discussion

This study identified a developmental peak of proliferative OPCs in mouse V1. 3-day birth-dating experiments using BrdU revealed that P22-25 is the sensitive period of proliferative OPC formation for BE at the time of eye opening and identified the bottom lamina of V1 (lower layer 6) as the sensitive site. BE elevated proliferative OPCs and lowered differentiation at the peak age. We identified the Shh signaling as a possible mechanism for the BE-induced elevation of proliferative OPCs. At P30, the P22-25 BrdU-labeled proliferative OPCs mostly exited the cell cycle and became quiescent 5 days later, employing a symmetric mode of cell segregation. BE did not affect this transition to quiescence. At P50, BE increased P22-25 BrdU-labeled, CNPase-expressing maturing OLs in the bottom lamina, employing the asymmetric and symmetric modes of cell segregation. Indeed, CNPase+ cell density was increased by BE among both BrdU-labeled and unlabeled cells. These data suggest that BE at the sensitive period of V1 development delays OPC differentiation but later promotes differentiation toward myelin formation. Here, we will discuss about the developmental peak of the proliferative OPCs, the BE-induced upregulation of the proliferative OPCs, the progression to quiescence of P22-25 proliferated OPCs, and their maturation at P50. Lastly, we will discuss limitations of this study.

Proliferative OPCs in V1 were elevated at P25, which corresponds roughly to the peak of ODP (reviewed in [49]). The 3-day BrdU labeling along with Ki67 immunostaining of OPCs for 3 consecutive periods over 9 days from P19 to P28 during the critical period of ODP revealed that the proliferative OPCs were elevated for P22-25 BrdU+ OPCs, while Ki67-lacking BrdU-labeled cells remained at the similar levels. Similar proliferation rates suggest a steady supply of differentiated cells into the gray matter during the critical period of ODP, while accumulating proliferative OPCs transiently for the peak. Though the relationship between ODP and the accumulation of the proliferative OPCs is unclear, the elevated proliferative OPCs at P25 may reflect the nature of the parenchyma during the important period of V1 development. Developmental gene expression analysis showed that oligodendrocyte and myelination-related genes were greatly elevated on P28 [17] and the proportion of gene expression was much higher for oligodendrocyte-related genes than neuron- or astrocyte-related genes on P26 [18]. Thus, the critical period of ODP, especially around its peak, may be a developmentally sensitive temporal window for OPC state changes and OL maturation.

Our present study demonstrated previously unknown effects of sensory experiences on OPCs in V1. Whisker lesion at birth, i.e., before the barrel formation around P4 [50], elevated proliferation and shifted the distribution of OPCs from the septa to the hollow of the barrel structure in the middle layer of the somatosensory cortex [13]. Whisker clipping at P6, i.e., at the beginning of cortical myelination, reduced differentiation and promoted proliferation as well as apoptosis after 4–6 days in the barrel field [14]. Thus, sensory loss shifted OPC states toward an immature state. Our observation of visual loss-induced delay toward differentiation at P25 is in line with the shift to an immature state. However, the upregulation of the proliferative OPC state by BE occurred without changes in the proliferation rate, in the absence of apoptosis, and in lower layer 6, distinguishing the behavior of OPCs between the two sensory deprivations. In the visual cortex, NG2 cell (presumptive OPC) densities developmentally decreased in layer 4 compared to layers 2/3 or layer 5 during the fourth postnatal week [13]. It was suggested that OPC cell proliferation or differentiation depended on the maturation of thalamocortical inputs. Mangin et al. (2012) reported that dark rearing from birth had no effects on OPC densities in layer 4 as well as layers 2/3 and layer 5. We also found no significant effects of visual loss on OPC densities in those layers. Although it is unclear whether dark rearing influences layer 6, our finding in the bottom lamina of V1 revealed a new phenomenon for sensory dependent effects on OPC development.

What underlies the sensitivity of the bottom layer to BE for OPC state changes needs consideration. One possibility would be local release of a factor(s) from laminar-specifically distributed cells. Astrocytes are well recognized to regulate OPC proliferation and differentiation (reviewed in [51–54]). Notably, developmental GFAP expression is restricted to layer 6 as well as in layers 1 and 2 on P24 following its expression throughout all layers on P12 [55]. Concomitant timing of the astrocyte upregulation and the proliferative peak of OPCs raises a possibility of a common factor(s) participating in their regulation in lower layer 6 around P25. One such factor may be Shh. A recent report shows that the cortical level of Shh transcripts and the N-terminal fragment of Shh protein gradually increased postnatally, plateauing around P21 and continuing to express at least until P30 [56]. Shh is expressed in NeuN-expressing cortical neurons [57] and in the bottom layer 5 neurons of the somatosensory cortex [58], where released Shh participates in synapse formation in the deep cortical layers via mature astrocytes during the fourth postnatal week [59]. Shh stimulates astrocytes to determine their phenotypes in developing brains [60]. When stimulated, astrocytes may regulate OPC proliferative states via growth factors like PDGF and FGF [61, 62] or regulate the timing of OPC differentiation via LIF [63, 64]. Local release of these or other factors via astrocytes may be involved in the Shh-dependent OPC state regulation we found in the present study.

The BE-induced upregulation of proliferative OPCs in lower layer 6 could also involve reorganization of neuronal circuits. It is unclear how BE at the time of eye-opening influences neuronal circuits in V1 at this point. Enucleation severs afferents to dorsal lateral geniculate nucleus (dLGN) and superior colliculus (SC), inducing degeneration of retinogeniculate axons [65]. Enucleation up to 15 days after birth causes adaptive changes in the visual and other systems [66]. BE at birth results in a reduced size of dLGN [67, 68] and a novel innervation of dLGN by the cuneate nucleus in mice [68]. dLGN also receives heteromodal axonal input from inferior colliculus (IC) in enucleated mole rats [69, 70] and hamsters [71]. Although dLGN innervation by IC neurons has not yet been reported in enucleated mice [72], such innervation has been observed in anophthalamic mice [72–75]. IC might also directly project to the bottom layers of V1 [74]. If these crossmodal reorganization occurs in our mouse model, non-visual stimuli could evoke functionally neuronal activities in V1 via dLGN, as has been observed in anophthalamic mice [74] and enucleated opossum [76]. It should be noted that thalamocortical projections to V1 was unaffected by BE or congenital blindness [77]. Since dLGN neurons project their axons throughout V1 with the main input to layer 4 and, to a lesser extent, layer 6 [78, 79], non-visual sensory activation may influence thalamic axon terminating areas in V1, especially where thalamocortical axon inputs activate locally-distributed OPC effector cells that release Shh. In addition to neurons, other possible sources of Shh would include OPCs themselves and astrocytes, which could produce Shh under some circumstances. In a demyelination model, Shh transcripts were highly expressed in oligodendrocyte lineage cells, and Shh overexpression increased the number of OPCs and mature OLs [80]. Meanwhile, astrocytes could reportedly release Shh postnatally after brain injuries [81–83]. Thus, a possible scenario for the elevation of proliferative OPCs at the bottom lamina might be then due to enhanced activation of the lower layer 6 due to reorganization of neural circuits and subsequent release of Shh from neurons or glial cells.

We found that P22-25 proliferated OPCs transitioned almost entirely to quiescent or differentiated cells over the 5 days from P25 to P30. The proliferated cells at P25 were about 80% Ki67-, suggesting most of them already exiting the cell cycle. Co-staining with Nestin and Ki67 suggested about one-third of the BrdU+ cells were presumably quiescent OPCs, and about a half of them were those differentiated or committed for differentiation. NG2 and Nestin co-staining confirmed that about a half of NG2+ cells were Nestin- differentiated cells. Since no CNPase+ cells were found among the BrdU+ cell at P25, however, we identified those Nestin- cells as pre-OLs. From P25 to P30, the pre-OL cell densities remain unchanged, while the proliferative OPCs among the P22-25 BrdU+ cells found at P25 (~20% for control, ~40% for BE mice) exited the cell cycle. We interpreted this result as the formation of quiescent cells based on the observation that pre-OLs did not increase from P25 to P30 and CNPase+ cells were few even at P30. The transition toward quiescence appears to involve Shh signaling since the continuous presence of cyclopamine during the transition period blocked Nestin+ undifferentiated cells from being upregulated by BE. This would be the first evidence for the role of Shh signaling on the OPC undifferentiation in the gray matter of the neocortex. Future studies will be necessary to determine molecular mechanisms for quiescent OPC formation.

Next, we will discuss potential consequences of the BE-induced upregulation of proliferative OPCs on differentiation and maturation. Some of the P22-25 BrdU-labeled pre-OLs expressed CNPase 5 days later at P30, where only 5% of doublets were asymmetrically-divided CNPase-expressing cells. With more extended time of 25 days, this percentage increased to ~20%, and symmetrically-divided CNPase+ doublets reached ~12.5% of doublets. This gradual process of developmental maturation is consistent with a previous work in the somatosensory cortex [14]. Previously, dark rearing did not alter the expression of MBP in V1 after the end of the critical period of ODP (P32) [20]. Their finding might be related to our observation at P30, where CNPase+ cell density or intensity was not affected largely by BE. Interestingly, after 25 days at P50, BE greatly increased CNPase-expressing BrdU+ doublets to nearly 90% of remaining doublets. BE increased not only the cell density of BrdU-labeled CNPase+ cells about 2-fold but also CNPase expression more than 60% in the bottom cortical lamina. These findings were unexpected given previous reports indicating that increased, rather than decreased, neuronal activities increased myelination in the cortical gray matter [8, 11, 12]. Our surprising observation of the increased differentiation and maturation in BE mice might involve neuronal circuit reorganization, recruiting other non-visual sensory inputs.

What underlies the transition of quiescent OPCs to maturation will require further investigation. A clue to this may come from studies in response to demyelination, where differentiation is enhanced with a delay following proliferation [84–87]. Possible mechanisms of promoted differentiation included activation of Nkx2.2 and Olig2 [86], Sox2 [88, 89], and Tcf7L2 via Wnt/β-catenin signaling [90, 91]. Regardless of the mechanism, BE affected differentiation after the peak of myelination (i.e., around third postnatal week; [92]). BE at the time of eye opening may have altered intrinsic mechanisms of OPC homeostasis from P22 to P25, elevating proliferative OPCs to be utilized later to increase OLs for possible adaptation to BE-induced plasticity involving neuronal excitability or neuronal circuit reorganization in layer 6.

Lastly, limitations of this work need to be discussed. First, the potential mechanisms underlying BE-induced OPC state changes will require further investigation. In this work, we have used in vivo injection of only one Shh signaling inhibitor cyclopamine. Additional experiments using other inhibitors of Shh signaling pathways or transgenic animals lacking a signaling molecule along the signaling pathways in specific cell types will be necessary to confirm the direct involvement of Shh signaling in OPCs and to reveal intracellular signaling mechanisms downstream to the Shh activation of its receptor. Second, additional evidence demonstrating the BE-induced shift toward undifferentiation will be important. We identified undifferentiated, quiescent, and differentiating cells using Ki67 and nestin. We used NG2 and nestin to differentiate pre-OLs and OPCs. More work will be necessary to not only characterize the precise relationship between OPC states and the expression of Ki67 or nestin but also confirm the state changes using other cell state-specific markers along with oligodendrocyte lineage makers. Third, the BE-induced increase in mature oligodendrocyte formation at later ages and the potential increase in myelination will need to be confirmed. We used CNPase as a sole marker for mature oligodendrocytes. The use of other markers will help to confirm the increase in mature OLs. Further, the confirmation of the potential increase in myelin and the identification of its target axons will be important to show the actual engagement of the upregulated OLs. It will be interesting to see which neuron type(s) (i.e., corticofugal or corticopetal projection neurons, and/or inhibitory neurons) were the target of the P22-25 born-OLs. Last, the relationship between BE-induced putative changes in neuronal activity and OPC development needs to be clarified. The sensitivity of BE on OPCs specifically in the bottom layer 6 is peculiar. Future studies will investigate potential changes in neuronal properties in the bottom lamina and their relationship with the extent of myelination.
